# A Model for Community-Based Pediatric Oral Heath: Implementation of an Infant Oral Care Program

**DOI:** 10.1155/2014/156821

**Published:** 2014-01-23

**Authors:** Francisco J. Ramos-Gomez

**Affiliations:** UCLA School of Dentistry, 10833 LeConte Ave, Los Angeles, CA 90048, USA

## Abstract

The Affordable Care Act (ACA) mandates risk assessments, preventive care, and evaluations based on outcomes. ACA compliance will require easily accessible, cost-effective care models that are flexible and simple to establish. UCLA has developed an Infant Oral Care Program (IOCP) in partnership with community-based organizations that is an intervention model providing culturally competent perinatal and infant oral care for underserved, low-income, and/or minority children aged 0–5 and their caregivers. In collaboration with the Venice Family Clinic's Simms/Mann Health and Wellness Center, UCLA Pediatrics, Women, Infants, and Children (WIC), and Early Head Start and Head Start programs, the IOCP increases family-centered care access and promotes early utilization of dental services in nontraditional, primary care settings. Emphasizing disease prevention, management, and care that is sensitive to cultural, language, and oral health literacy challenges, IOCP patients achieve better oral health maintenance “in health” not in “disease modality”. IOCP uses interprofessional education to promote pediatric oral health across multiple disciplines and highlights the necessity for the “age-one visit”. This innovative clinical model facilitates early intervention and disease management. It sets a new standard of minimally invasive dental care that is widely available and prevention focused, with high retention rates due to strong collaborations with the community-based organizations serving these vulnerable, high-risk children.

## 1. Introduction

The US Surgeon General has identified early childhood caries (ECC) as the most common chronic childhood disease; it is five times more prevalent than asthma [1]. It is a highly infectious disease caused by bacteria easily transmitted horizontally from person to person and vertically from caregiver to child. As a result, even newborns are susceptible to infection [2].

About 80% of dental disease, including ECC, is concentrated in 20%–25% of children, primarily those from low-income and/or minority backgrounds [3, 4]. Ironically, those at highest risk are also those who face the greatest barriers to accessing early and ongoing dental care [5, 6]. Approximately 25% of children younger than six years of age have seen a dentist with the probability decreasing based on lower levels of income [5]. While many of these children are hindered in obtaining dental care by their socioeconomic level, ethnicity, primary language, and the education level of their parents or caregivers, many families also only seek care when a problem arises or permanent teeth have erupted. Preventive care is neither a priority nor deemed essential. Even among those with insurance, utilization rates are low, particularly in public insurance programs. In fact, the pediatric dental service utilization rate for the USA's largest safety net program, Medicaid, is only 38% [7].

Although ECC is exceedingly prevalent among young children, it is also highly preventable with early intervention. Early identification of oral diseases like ECC can reduce the risk of, arrest, or even reverse disease. The American Academy of Pediatric Dentistry [8, 9], American Dental Association [10], American Academy of Pediatrics [11], American Association of Public Health Dentistry, [12], and many public health organizations have recommended that children be seen by a dentist on a recurring basis no later than six months after the eruption of a child's first tooth or by one year of age. Although this protocol was adopted 27 years ago, dental professionals have also not wholeheartedly endorsed the recommendation and many parents and caregivers remain unaware of this advice.

As such, strategies are necessary to ensure and promote early recurring dental care, particularly for populations suffering the greatest burden of disease. To that end, for instance, the American Academy of Pediatric Dentists has funded grants to improve access to high quality dental care for children in need. From 2010 to 2012, eighteen grant recipients received assistance to support community-based programs that expanded dental care to children in need; another 15 received grants in 2013 [13]. The American Dental Association launched its Give Kids A Smile (GKAS) program in 2003 to encourage dentists nationwide to provide free dental care to underserved children. Through GKAS, dentists volunteer their time and services year-round through local health fairs and other events [14]. Other options include free or low-cost programs through dental school clinics, programs offered through state or local health departments, or at non-profit organizations including school-based health centers [15, 16]. In many programs, patient retention rates are often low for reasons that include housing and employment instability, lack of transportation, and unreliable communication methods. However, despite the success of these grants and programs, the need for consistent, ongoing dental care continues to grow. While these programs have assisted with serving more children in need, a greater focus on reaching children through untraditional venues, such as by engaging community stakeholders like Head Start/Early Head Start (HS/EHS), Women, Infants, and Children (WIC), day care centers, and schools, is required [17].

The Commission Dental Accreditation (CODA) has also recently recognized the value of community-based learning for dental students by updating their standards to include broader clinical experiences [18]. Most dental schools typically provide care through a number of venues, such as university and affiliated hospital clinics, mobile dental vans, and community-based health centers. UCLA has had a long standing commitment to providing services to underserved populations and has been a leader in recognizing the education value of diverse clinical encounters. Ahead of CODA's recent change in regulations, UCLA established its Community Health and Advocacy Postdoctoral Resident Training (CHAT) program in 2006, and in 2010, UCLA realized an opportunity to strengthen their focus on community-based care by partnering with key stakeholders to inaugurate UCLA's Infant Oral Care Program (IOCP) through the Pediatric Section of the School of Dentistry.

## 2. Materials and Methods

IOCP launched in 2010 through UCLA's School of Dentistry's Section of Pediatric Dentistry in partnership with the Venice Family Clinic's Simms/Mann Health and Wellness Center (VFC) and nearby WIC and EHS/HS sites. IOCP functions on the assumption that at-risk children and their parents/caregivers visit venues like community clinics and HS/EHS and WIC sites earlier and with more regularity than dental clinics. Therefore, these program sites offered significant promise as partners for outreach, education, and referrals to increase compliance with the age-one visit [19]. In some cases, these partners can also become venues through which care is provided, thereby increasing entry points to care and the opportunity to become children's dental homes; that is, a stable facility through which early, ongoing, and culturally sensitive dental care may be provided to children starting at perinatal stage through infancy. These collaborations foster an integrated approach to health care, where a team of dentists and nondental providers, such as pediatricians, nurse practitioners, and obstetricians, as well as community workers can cross-train to offer better care that improves both dental and overall health. This increases the quality of care both types of providers give and inevitably increases access to dental care for vulnerable populations and reduces oral health disparities.

IOCP was established with the goal of offering early and ongoing dental care to low-income and/or minority children aged 0–5 years old. VFC and WIC donated office space and medical exam rooms for IOCP operations. The IOCP provided trainings to all community partners, for example, WIC and HS/EHS staff, VFC pediatricians and nurse practitioners, and other allied health workers, on the effect of oral health on overall health over the life course. These trainings were key in obtaining patient recruitment and referrals as well as to initiate a cultural and perception change on when to seek dental care. Pediatric residents, supervised by faculty and assisted by 3rd and 4th year predoctoral dental students, conducted exams for IOCP patients. Exam protocols emphasized early, ongoing care provided in a culturally appropriate manner. The IOCP remained the child's dental home until he/she “graduated” at age 3–5 years to a full service dental clinic. In addition, patients of the IOCP who required restorations or other more invasive procedures were seamlessly referred to the VFC's dental clinic, while referrals for tertiary care, such as full mouth rehabilitation under general anesthesia, were made to university clinics or comparable hospital programs. Whenever possible, the IOCP designed its operational procedures to be as simple and as streamlined as possible, to make access entry and continuation effortless for its patients and their families.

### 2.1. Operational Model

IOCP has limited overhead and start-up costs. The provision of basic dental services only requires a “pod”—a private room with two chairs and a portable light as well as educational materials, intake forms, and disposable dental equipment and supplies such as mirrors, gloves, fluoride varnish, and gauze. For UCLA's IOCP, VFC and WIC provided space within their existing facilities for a minimum of four hours per week. With an already established pool of low-income and/or minority patients and clients at these facilities, the IOCP had immediate access to its target population; for example, VFC had a well-established Well Baby clinic. All IOCP patients were required to register as a patient of VFC to facilitate tracking, record keeping, and care coordination. Initially, IOCP clinicians did not have access to an electronic medical record system (EHR). Data and communications with caregivers and other providers were manually captured and tracked. However, the recent installation of new software at VFC has enabled the IOCP clinicians to more efficiently document and track a child's oral health status over time through electronic medical records (EHR), which have incorporated forms for caries management by risk assessment (CAMBRA) and self-management goals.

IOCP is also a required three-month rotation for UCLA pediatric dentistry residents to provide more in-depth exposure to working within a community health setting with children at high risk for disease due to socioeconomic circumstances. In addition, their IOCP rotation is supplemented by didactic experiences that enhance their understanding of oral health from a public health perspective, rather than merely clinical one. IOCP is also an elective offered to third and fourth year predoctoral dental students interested in increasing their experience in pediatric dentistry. Candidates in UCLA's Advanced Education in General Dentistry program and foreign-trained dentists participating in UCLA's Preceptorship program may also elect for a rotation through IOCP. Even further, practicing dentists of any type as well as pediatricians and nurse practitioners may participate in IOCP; in fact, many have taken part to increase their comfort level in working with children and their understanding of access disparities for high-risk populations.

All partner staff involved in IOCP received trainings led by UCLA pediatric dentistry residents on the oral disease process and commitment to oral health. To more deliberately encourage these diverse team members to work collaboratively, structured discussions are also held to gain consensus on how each profession can contribute to a child's optimal oral health and on how to better coordinate care across disciplines to improve their health through IOCP. Due to the cultural diversity of the patients served, IOCP practitioners also received specific training to sensitize them to the language, culture, and oral health literacy challenges they would face in order to effectively treat these patients. Further, a focus on interprofessional collaboration among medical and dental professionals and with community-based organizations required taking a multifaceted approach to “health”, including a focus on holistic and comprehensive care that factor in things that include but are not limited to diet and physical activity.

### 2.2. The Patient Visit and Risk-Based Care

The IOCP provides early and culturally competent perinatal and infant oral care for mothers/caregivers and children aged 0–5 years old based on a simple standard of care infant oral protocol [20]. The IOCP improves oral health outcomes through a disease prevention and management model focused on establishing a dental home and on an individualized, oral health risk-based schedule of recall visits. This complies with the recommendation of several national and medical professional organizations including the American Academy of Pediatric Dentistry [8, 9], American Dental Association [10], American Academy of Pediatrics [11], and American Association of Public Health Dentistry [12] for their “age-one visit” and the establishment of a dental home.

**Figure 1:**
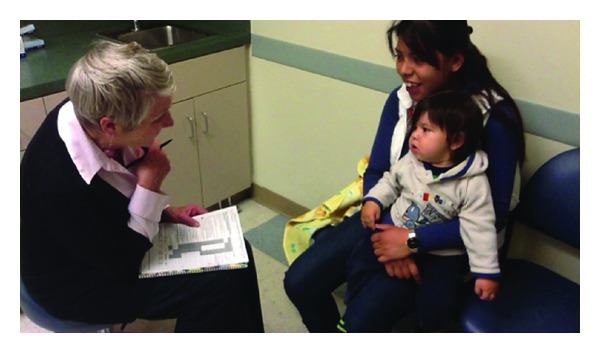
Step 1: CAMBRA interview.

At each scheduled visit, providers conduct an Infant Well Baby Oral Exam, similar to a Well Baby visit with a pediatrician. This exam includes six steps: (1) caries risk assessment, (2) proper positioning of the child for a knee-to-knee exam, (3) age-appropriate tooth-brushing prophylaxis, (4) a clinical exam, (5) fluoride varnish treatment, and (6) anticipatory guidance, counseling, and self-management goals [19]. The most critical step of the Infant Well Baby Oral Exam is the caries risk assessment. Conducted through an interview with the parent/caregiver, the caries risk assessment offers an opportunity to set visit expectations and establish rapport with the child and the caregiver (Figure 1). The examiner can also begin gathering key information on the child's risk and protective factors that, when combined with clinical findings, will be the foundation of a treatment plan based on the child's individual risk for developing caries.

At VFC, IOCP clinicians use the caries management By risk assessment (CAMBRA) caries risk assessment tool to rate a child as having high, moderate, or low caries risk (Figure 7). A child's caries risk level is used to design and implement a minimally invasive treatment plan, or “care path”, that factors a child's biology and individual, family, and community factors that can influence oral health such as prior cultural and country norms and fluoridation of public water supplies. CAMBRA guides clinicians to prevent and manage disease for their patients using a comprehensive approach that utilizes anticipatory guidance, counseling, and the creation of self-management goals tailored to the child's age and individual risk [21].

**Figure 2:**
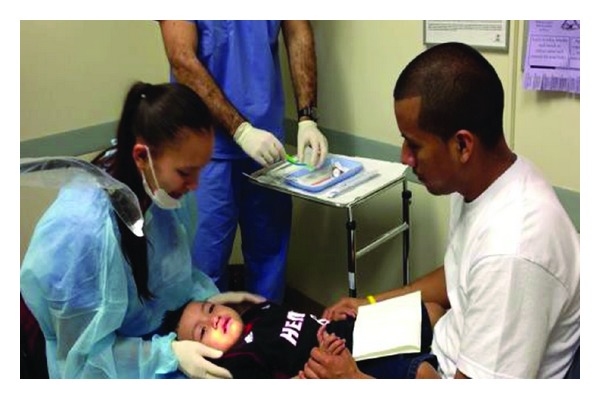
Step 2: knee-to-knee exam.

The CAMBRA interview is followed by the oral exam. First and foremost is proper positioning of the child to ensure that he/she is comfortable, safe, and secure. In young children or those with special needs, a knee-to-knee position is best (Figure 2). In the knee-to-knee position, the parent/caregiver sits facing the dental examiner and the child lays with his/her head in the examiner's lap. This allows the parent/caregiver to see the child's face and hold their child's hands in theirs, while maintaining control over the child's legs. In this position, the parent can also observe and learn about his/her child's teeth and development.

**Figure 3:**
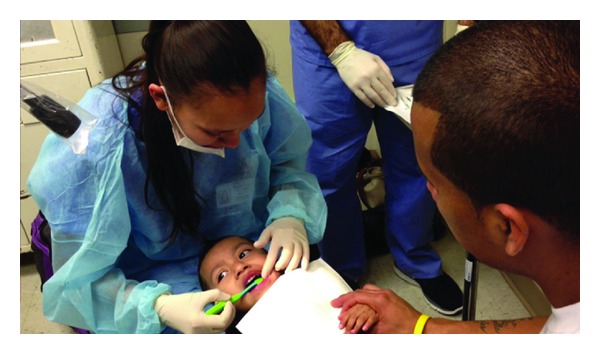
Step 3: toothbrush prophylaxis.

During each exam, the provider performs a toothbrush prophylaxis to remove any plaque or debris from the teeth prior to the clinical exam (Figure 3). Using the tell-show-do technique the examiner can also demonstrate the proper technique for brushing the child's teeth for the parent/caregiver.

**Figure 4:**
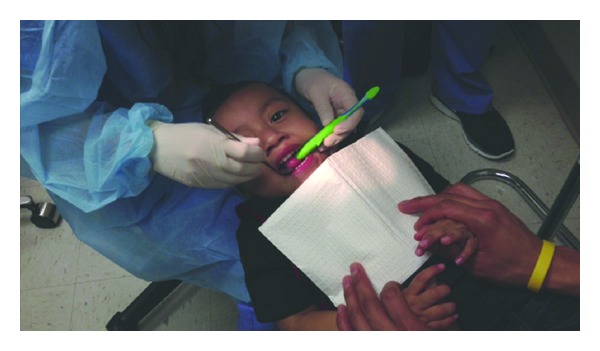
Step 4: clinical exam.

**Figure 5:**
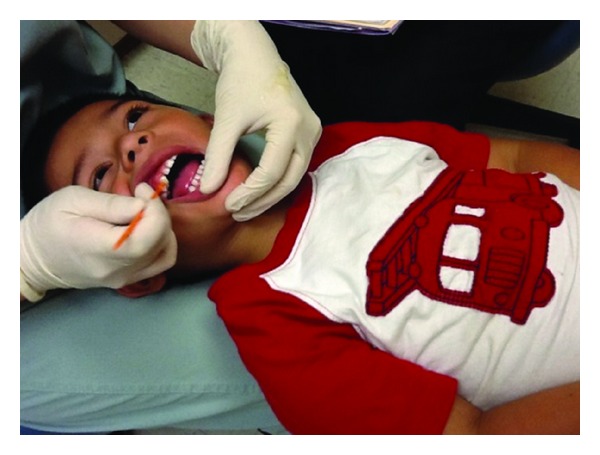
Step 5: fluoride varnish.

**Figure 6:**
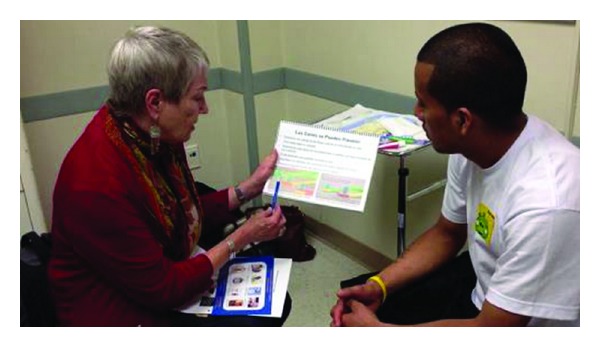
Step 6: self-management goals.

**Figure 7:**
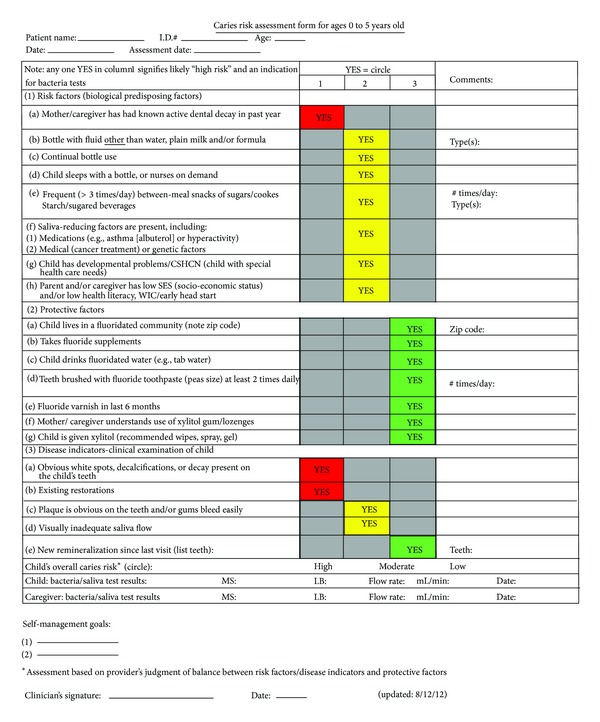
CAMBRA form.

**Figure 8:**
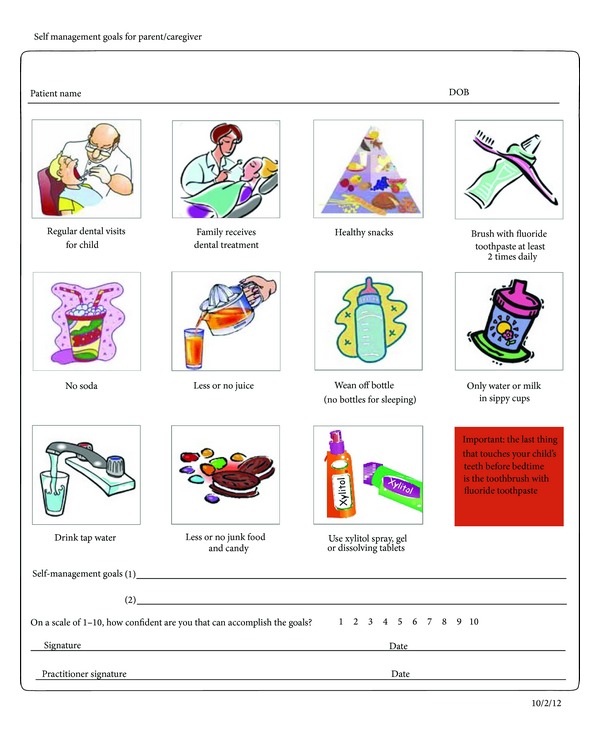
Self-management goals.

The examiner will then conduct a clinical exam that includes counting the child's teeth aloud, using the toothbrush handle as a mouth prop, if necessary (Figure 4). Since a child may start to fidget at this point, practitioners often make a game of this task, singing songs and so forth, to engage the child. Examiners should remember to praise the child often for his/her cooperation during the process. During the process of counting, the examiner should inspect the soft tissues, hard tissues, and occlusion of the child's mouth documenting any visible plaque and its location; white spot lesions; demineralized or remineralized enamel; brown spots on the occlusal surfaces; tooth defects; deep pits/fissures; missing and/or decayed teeth; existing restorations; defective restoration; gingivitis or other soft tissue abnormalities; occlusions; and any indications of trauma. The information from the clinical exam is then combined with data gathered during the CAMBRA interview to determine the child's individual caries risk as well as a care path that establishes the periodicity of follow-up visits. For example, while most children are recommended for a reapplication of fluoride varnish at a minimum of every six months, a monthly reapplication may be required in some children to reduce ECC risk. In addition, in children with severe ECC, topical fluoride may be insufficient alone to overcome a particular child's bacterial challenges. In this situation, additional interventions such as combination therapies based on age and risk, antibacterial regimens, or more frequent examinations may be used to arrest progression, protect the tooth structure, and implement measures to break the cycle of continued reinfection. Needs for acute or specialized care, such as restorative treatment, are referred out. After the clinical exam, fluoride varnish is applied to the child's teeth, consistent with current accepted prevention protocols (Figure 5). All children and caregivers also receive oral health education, which covers the causes, onset, and progression of oral disease. The establishment of self-management goals is the final step of the Infant Well Baby Oral Exam. (Figures 6 and 8). Integrated as part of the child's care path, parents/caregivers are asked to select two of the several recommended behavioral modifications proposed in Figure 8. For instance, a parent may need to improve upon their own and their children's oral hygiene practices, such as brushing at least twice daily using fluoridated toothpaste. Others may need to focus on reducing their children's intake of sugary foods and beverages, particularly before bedtime. Meanwhile, a caregiver may simply need to be reminded that regular checkups are necessary even when children do not have any pain or difficulty chewing.

## 3. Results

Although the program provides care only four hours per week at each site (VFC and WIC), IOCP has been able to reach a significant portion of its target population earlier than planned and with higher retention levels than have been seen in dental clinics. In fact, IOCP has attended to 672 unique patients across over 1,500 visits since its inception in 2010. Slightly more than 42% of the children in IOCP have had two or more visits, and the numbers continue to increase.

As of July 2013, among those patients who have not graduated to the VFC dental clinical, IOCP maintained 138 patients as caries-free and prevented precavitated lesions from progressing in 51 patients. These successes are attributed to capturing underserved populations through proactive referrals from our community partners and case management and triage based on individual risk and by interdisciplinary clinic staff. Part of the success may also be due to positive shifts in parental and caregiver knowledge and attitudes regarding oral health.

The quality improvement measures tracked include, but are not limited to, the following:

- Percent of ECC patients presenting with new cavitation;
- Percent of ECC patients presenting with pain from untreated decay;
- Percent of ECC patients with documented caries (high, medium, and low);
- Percent of ECC patients who had disease management visiting within the recommended interval based on risk;
- Percent of ECC patients with self-management goals reviewed at most recent disease management visit;
- Percent of ECC patients whose risk status has improved.

The above measures have helped isolate areas for improvement in IOCP and develop disease management and prevention strategies that can be implemented on a much wider scale. We believe that long-term analysis will provide evidence showing the efficacy of IOCP in reducing the burden of oral disease, developing a strong case for expanding similar oral health disease prevention and management programs elsewhere.

## 4. Discussion

The integration of oral health into primary medical care can improve the continuity of care between dental and medical homes and could foster better health behaviors that could achieve and preserve good oral health, resulting in a lower disease risk [22]. As the population grows and diversifies, the oral health disparities gap will widen. At the policy level, programs that service low-income and/or minority families should be strengthened. States not currently offering adult Medicaid dental benefits should be encouraged to offer dental services, at a minimum, to its pregnant beneficiaries to prevent the vertical transmission of disease.

Coordinated community outreach is also important. More must be done to achieve consensus and acceptance within the dental community on enforcing the age-1 visit recommendation. Medical personnel, especially pediatric and obstetric professionals and allied health workers, must understand the correlation between a mother's oral health status and its impact on her child(ren), and they must also endorse and promote the age-1 visit to their patients. Community-based organizations, such as Head Start/Early Head Start (HS/EHS), Women, Infants, and Children (WIC), day care centers, and schools need to be actively engaged in educating their parent and caregiver participants on the need for regular dental checkups beginning at the age of 1. Finally, cost-effective, easily accessible, family-centered, culturally sensitive models of care are needed. The success of an Infant Oral Care Program depends on overcoming such challenges.

The IOCP is also built upon the principle that prevention of oral disease is preferable to surgical treatment. The IOCP emphasizes the need for risk assessments so that care can be tailored to the individual child's need as opposed to a one-size-fits-all approach to recall visits, fluoride varnish applications, and other preventive care. This concept, while not new, is often difficult to promote since, in many states, more traditional dental treatments generate income, while the cost effectiveness of prevention is harder to enumerate. In addition, Medicaid reimbursement rates may not cover all the activities recommended by the IOCP. Sites with existing dental clinics may see the IOCP as a program that could decrease revenue. However, the IOCP is intended to maximize resource utilization by increasing the number of patients overall for the clinic with only the more acute cases necessitating more expensive clinical chair time and where net dental home patients also increase. Therefore, mandates to change to reimbursement rates are also needed to incent dental providers to increase their acceptance of Medicaid patients and shift emphasis to preventing disease.

Dental programs need to act now to update their curriculum to provide future dentists with a skill set that can address the growing community need and provide their doctoral candidates with the opportunity to gain proficiency through community practice. Cultural sensitivity will become more critical and dental schools also need to incorporate risk assessment and preventive care programs with an emphasis equal to cavitation treatment options.

The IOCP is an easily replicable program with low start-up and maintenance costs. However, success is contingent upon establishing strong community-based partnerships in order to overcome the many biological, behavioral, and environmental differences among vulnerable populations that influence health outcomes. IOCP community partnerships were selected based on each program's close proximity to the IOCP operational site. WIC and EHS/HS were chosen because their program dynamics had a retention element already established, for example, appointments to pick up vouchers, attendance of their children, and so forth. In fact, any site can establish an IOCP and since most families accept a periodicity schedule for infant and toddler health care exams and procedures, such as immunizations, dental home visits, for example, can be offered on the same day and at the same venue as nondental appointments like Well-Baby visits. This strategy, combined with outreach and services provided by organizations similar to Early Head Start, Head Start, and WIC, can facilitate and has facilitated access to culturally sensitive oral health care screening, education and services for low-income and vulnerable populations by simplifying the entry process and linking it to other services that they are already utilizing with regularity.

## 5. Conclusion

IOCP effectively coalesces a multidisciplinary care team to establish a model for a new generation of healthcare providers and social service staff, all of whom will have the capacity to address the oral health needs for patients of all ages and backgrounds. Subsequently, this could reduce current disparities in oral health care access and disease among vulnerable populations that include children and low-income and/or minority families. Programs similar to the IOCP are important to the future of dental care to increase entry points for accessing care and to provide appropriate training for general dentists and other pediatric providers. Designed to complement existing medical and dental primary clinical settings, the IOCP provides a low-cost alternative to providing a dental home to a young population of children prior to the onset of dental disease which may require intervention in a full clinical environment. There is evidence based results on the success of utilizing community-based, social service partnerships in close proximity to the proposed operational site to increase patient early age recruitment and retention in a disease prevention management model such as the IOCP. The IOCP also importantly functions as a training opportunity for both dental and nondental professionals to increase experience, training, proficiency, and acceptance in treating very young children, aged 0–5 years, and keep their healthy teeth healthier.

However, multidisciplinary collaboration is not enough; as noted, care must also be culturally sensitive as critical factor in care. Professionals, both dental and nondental, need to begin to understand the importance of achieving and maintaining good oral health as an integral part of total health in order to address the emerging oral health crisis. To prepare for these changes, dentists and the providers with whom they collaborate will need to know how to best serve their patients using an individualized, age-appropriate, and risk-based approach to care and practice applying their knowledge in the community.

## References

[1] US Department of Health and Human Services (2000). Oral health in America. *A Report of the Surgeon General*.

[2] Ramos-Gomez FJ, Gansky SA, Featherstone JDB (2012). Mother and youth access (MAYA) maternal chlorhexidine, counselling and paediatric fluoride varnish randomized clinical trial to prevent early childhood caries. *International Journal of Paediatric Dentistry*.

[3] Center for Oral Health Mommy, it hurts to chew: the California smile survey, an oral health assessment of California’s kindergarten and 3rd grade children. http://www.centerfororalhealth.org/images/lib_PDF/dhf_2006_report.pdf.

[4] Liu J, Probst JC, Martin AB, Wang J-Y, Salinas CF (2007). Disparities in dental insurance coverage and dental care among US children: the national survey of children’s health. *Pediatrics*.

[5] Edelstein BL, Chinn CH (2009). Update on disparities in oral health and access to dental care for America’s children. *Academic Pediatrics*.

[6] Crall JJ (2005). Development and integration of oral health services for preschool-age children. *Pediatric Dentistry*.

[7] Centers for Medicare and Medicaid Services Medicaid/CHIP oral health services. http://www.medicaid.gov/Medicaid-CHIP-Program-Information/By-Topics/Benefits/Downloads/2010-Dental-Factsheet.pdf.

[8] American Academy of Pediatric Dentistry Council on Clinical Affairs (2005). Policy on the dental home. *Pediatric Dentistry*.

[9] American Academy of Pediatric Dentists Get it done in year one. http://www.aapd.org/assets/2/7/GetItDoneInYearOne.pdf.

[10] American Dental Association Statement on early childhood caries. http://www.ada.org/2057.aspx.

[11] American Academy of Pediatrics http://www2.aap.org/oralhealth/AboutUs.html.

[12] American Association of Public Health Dentistry First oral health assessment policy. http://www.aaphd.org/default.asp?page=FirstHealthPolicy.htm.

[13] American Academy of Pediatrics Getting children dental care they need. http://www.aapd.org/foundation/kids/.

[14] American Dental Association http://www.ada.org/givekidsasmile.aspx.

[15] National Institute of Dental and Craniofacial Research Finding low-cost dental care. http://www.nidcr.nih.gov/oralhealth/popularpublications/findinglowcostdentalcare/.

[16] Health Resources and Services Administration Oral health: women & children. http://www.hrsa.gov/publichealth/clinical/oralhealth/maternalchild.html.

[17] Mouradian WE (2006). Band-aid solutions to the dental access crisis: conceptually flawed—a response to Dr. David H. Smith. *Journal of Dental Education*.

[18] Mathieson KM, Gross-Panico ML, Cottam WW, Woldt JL (2013). Critical incidents, successes, and challenges of community-based dental education. *Journal of Dental Education*.

[19] Kaiser Family Foundation Filling an urgent need: improving children’s access to dental care in Medicaid and SCHIP. http://kaiserfamilyfoundation.files.wordpress.com/2013/01/7792.pdf.

[20] Ramos-Gomez FJ, Ng MW (2009). Six step protocol for a successful infant oral care visit. *Pediatric Dentistry Today*.

[21] Ramos-Gomez FJ, Crall J, Gansky SA, Slayton RL, Featherstone JDB (2007). Caries risk assessment appropriate for the age 1 visit (infants and toddlers). *Journal of the California Dental Association*.

[22] Mouradian WE, Wehr E, Crall JJ (2000). Disparities in children’s oral health and access to dental care. *Journal of the American Medical Association*.

